# Effects of different combinations of radical nephroureterectomy and bladder cuff excision procedures for upper tract urothelial carcinoma on bladder recurrence

**DOI:** 10.1590/S1677-5538.IBJU.2023.0031

**Published:** 2023-05-20

**Authors:** Eric Yi-Hsiu Huang, Meng-Che Tai, Hsiao-Jen Chung, Yen-Hwa Chang, William J. Huang

**Affiliations:** 1 Taipei Veterans General Hospital Department of Urology Taipei Taiwan Department of Urology, Taipei Veterans General Hospital, Taipei, Taiwan; 2 National Yang Ming Chiao Tung University College of Medicine and Shu-Tien Urological Research Center Department of Urology Taipei Taiwan Department of Urology, College of Medicine and Shu-Tien Urological Research Center, National Yang Ming Chiao Tung University, Taipei, Taiwan; 3 Taipei Veterans General Hospital Taoyuan Branch Department of Surgery Division of Urology Taoyuan Taiwan Division of Urology, Department of Surgery, Taipei Veterans General Hospital Taoyuan Branch, Taoyuan, Taiwan

**Keywords:** Nephroureterectomy, Urinary Bladder, Recurrence

## Abstract

**Purpose:**

To compare the effects of different combinations of radical nephroureterectomy (RNU) and bladder cuff excision (BCE) surgical procedures on intravesical recurrence (IVR) in patients with upper tract urothelial carcinoma (UTUC).

**Materials and Methods:**

This retrospective observational study included 452 patients who underwent RNU with BCE for UTUC between January 2010 and December 2020. The patients were classified into three groups based on different combinations of RNU and BCE surgical procedures: open RNU with open BCE (group 1, n=104), minimally invasive (MIS) RNU with open BCE (group 2, n=196), and MIS RNU with intracorporeal BCE (group 3, n=152). Data on demographics, body mass index, history, preoperative renal function, perioperative status, tumor characteristics, histopathology, and recurrence conditions were collected. Multivariate Cox regression analyses were performed to determine the impact of the surgical procedures on IVR. P-values <0.05 were considered statistically significant.

**Results:**

After a median follow-up of 29.5 months, the IVR rate was 29.6% and the IVR-free survival rate was the lowest in group 2 (group 1 vs. group 2 vs. group 3: 69.0% vs. 55.1% vs. 67.5%; log-rank P=0.048). The overall survival rate was comparable among the three groups. Multivariate analysis revealed that group 2 had a significantly higher risk of IVR than group 1 (hazard ratio=1.949, 95% confidence interval=1.082–3.511, P=0.026), while groups 1 and 3 had similar risks.

**Conclusions:**

For patients with UTUC, MIS RNU with open BCE is associated with a higher risk of IVR than open RNU with open BCE and MIS RNU with intracorporeal BCE.

## INTRODUCTION

Radical nephroureterectomy (RNU) with complete bladder cuff excision (BCE) is the standard treatment for high-risk upper tract urothelial carcinoma (UTUC). The European Association of Urology guidelines state that there is a tendency towards equivalent oncological outcomes between laparoscopic and open RNU. Recent data show that a robot-assisted laparoscopic approach can achieve oncologic equivalence with other approaches (1). With respect to BCE, several techniques have been described, including open excision, transurethral resection of the ureteral orifice, ureteric intussusception, and pure laparoscopy or pure robotic approaches (2). The oncological outcomes of these approaches are conflicting (3, 4). Many urologists combine laparoscopic or robotic RNU with open BCE via a lower abdominal incision. Alternatively, several completely minimally invasive procedures have also been reported to be feasible and safe, such as transperitoneal laparoscopic RNU with transurethral endoscopic BCE (5), pure retroperitoneal laparoscopic RNU with BCE (6), and completely retroperitoneal robot assisted RNU with BCE (7). There are various surgical combinations of RNU and BCE. However, few studies have evaluated the oncological outcomes of the different combinations.

Intravesical recurrence (IVR) after RNU BCE occurs in as high as 22–47% of patients (1, 8–12). IVR usually occurs within 2 years following the treatment of UTUC (13). Seisen et al. conducted a meta-analysis on IVR after RNU for UTUC and found that patient-, tumor-, and surgery-specific factors were predictors of IVR (14). The surgery-specific predictors of IVR included the laparoscopic approach, extravesical bladder cuff removal, and positive surgical margins.

However, besides the previously identified predictors of IVR after RNU BCE, other surgery-specific factors (e.g., different combinations of RNU and BCE surgical procedures) may also play an important role in IVR, but these have not been previously discussed. We hypothesized that different surgical combinations of RNU and BCE may have effects on the IVR rate after RNU and BCE. Therefore, this study aimed to compare the effects of different combinations of RNU and BCE surgical procedures on IVR in patients with UTUC.

## MATERIALS AND METHODS

### Study design and subjects

This retrospective observational study was approved by our Institutional Review Board (2020-12-007BC). The requirement for informed consent was waived due to the retrospective nature of the study. Patients who underwent RNU BCE for UTUC between January 2010 and December 2020 at our hospital were included. The exclusion criteria were as follows: a history of bladder cancer, concomitant bladder cancer, bilateral UTUC, UC in the graft kidney, distant metastasis at diagnosis, and neoadjuvant systemic therapy for UTUC up to 6 months prior to surgery. All patients were suspected to have upper urinary tract tumors and underwent preoperative imaging studies including sonography, computed tomography (CT) urography, and magnetic resonance imaging. The diagnosis was eventually confirmed by ureteroscopic biopsy within 1 month before RNU BCE in all the patients, and concomitant bladder cancers were identified if present. The surgical procedures for RNU BCE were decided by the operating surgeons. Among the 723 patients who met the inclusion criteria, 271 patients were excluded. The 452 patients included in the analysis were classified into three groups according to the surgical procedure combination: open RNU with open BCE (group 1), Minimally invasive (MIS) RNU with open BCE (group 2), and MIS RNU with intracorporeal BCE (group 3).

### Surgical Procedures

#### Open RNU with open BCE

A midline incision was created, and the peritoneal cavity was entered. The colon was medially reflected. BCE was performed first in an extravesical fashion after the lower ureter was identified. The urinary bladder defect was closed after the completion of BCE. Dissection was conducted toward the renal hilum, and RNU was completed after controlling the renal pedicles.

#### MIS RNU with open BCE

The MIS RNU utilized either laparoscopic or robotic RNU. Laparoscopic RNU was performed with the renal pedicle controlled by a laparoscopic endovascular stapler. The ureters were secured with a Hem-o-lok (Teleflex, Wayne, PA, USA) after laparoscopic endovascular stapler application. Once laparoscopic RNU was completed, open BCE was performed extravesically and the urinary bladder defect was closed with sutures.

Robotic RNU was performed with either the da Vinci Si or Xi surgical systems (Intuitive Surgical, Sunnyvale, CA, USA). The robotic RNU procedure was similar to the laparoscopic RNU. Once the robotic RNU component of the surgery was completed, open BCE was performed in the same manner as that for the laparoscopic procedure.

#### MIS RNU with intracorporeal BCE

The MIS RNU was performed using either laparoscopy or robotics, as previously described. Once RNU was completed, extravesical BCE with MIS was performed, and the urinary bladder defect was closed intracorporeally.

#### Postoperative follow-up

All patients underwent regular postoperative follow-ups, including patient history and physical examination, urinalysis, urine cytology, chest radiography, abdominal ultrasonography or CT, and cystoscopy. Cystoscopy was performed every 3 months for the first 2 years, every 6 months for the following 2 years, and annually thereafter. Abdominal ultrasonography or CT was performed every 6-12 months or when clinically indicated. Systemic adjuvant therapy was administered to patients with locally advanced disease. IVR was defined as the presence of a bladder tumor during cystoscopy follow-up and confirmed by biopsy.

#### Data collection

All patient data were retrospectively collected through a review of the medical records. Variables included demographics (sex and age), body mass index (BMI), previous medical history, preoperative renal function (serum creatinine), hydronephrosis, perioperative data (operation time and estimated blood loss), tumor characteristics, and recurrence condition.

Tumor characteristics included laterality, site, histological type, stage, grade, bladder cuff involvement, variant histology, lymphovascular invasion, surgical margin, and lymph node metastasis. Pathological staging was based on the 2009 American Joint Committee on Cancer TNM classification system (15), and tumors were graded according to the 2004 World Health Organization classiﬁcation (16). Variant histology included any bladder malignancy other than pure urothelial cancer, such as squamous differentiation, sarcomatoid change, micropapillary pattern, glandular differentiation, lymphoepithelioma-like carcinoma variant, and mixed small-cell neuroendocrine carcinoma.

Recurrence data included the IVR status, time to IVR, and adjuvant treatment before IVR.

### Statistical Analysis

Data are expressed as the mean ± standard deviation (SD) for continuous variables and as counts with percentages for categorical variables. Comparisons were made using the one-way analysis of variance for continuous variables and chi-square test for categorical variables. The time to IVR was estimated using the Kaplan–Meier method and compared between groups using the log-rank test. Cox regression analyses were performed to determine the impact of surgical procedures on IVR. Variables with P <0.1 in the univariate model were included in the multivariate model. The results of Cox regression analysis are presented as hazard ratios (HR) with 95% CI. All statistical analyses were performed using SPSS software version 24 (IBM Corp., Armonk, NY, USA). Statistical significance was set at a P-value of <0.05.

## RESULTS

The mean patient age was 71.9±10.3 years, and 208 (46.0%) patients were male. There were 104, 196, and 152 patients in group 1, group 2, and group 3, respectively. The demographics, baseline characteristics, perioperative data, tumor characteristics, and recurrence conditions were comparable among the three groups, except that group 1 had significantly greater blood loss (P<0.001) (Table-1).

**Table 1:** Comparison of demographics, baseline characteristics, perioperative data, and tumor characteristics among the three groups.

Variables		Group 1	Group 2	Group 3	P-value
Surgical procedure		Open RNU + open BCE	MIS RNU + open BCE	MIS RNU + intracorporeal BCE	
Number		104	196	152	
Age (year)		70.4 ± 11.1	71.9 ± 10.5	72.9 ± 9.4	0.161
Male		46 (44.2%)	87 (44.4%)	75 (49.3%)	0.601
BMI (kg/m^2^)		25.0 ± 3.9	24.5 ± 3.8	24.5 ± 4.0	0.493
HTN		63 (60.6%)	100 (51.0%)	70 (46.1%)	0.072
DM		22 (21.2%)	60 (30.6%)	39 (25.7%)	0.197
CAD		8 (7.7%)	28 (14.3%)	20 (13.2%)	0.241
Smoking*		20 (19.2%)	34 (17.3%)	27 (17.8%)	0.920
**Symptoms**					**0.523**
	Hematuria	90 (86.5%)	153 (78.1%)	122 (80.2%)	
	Other symptoms	5 (4.8%)	14 (7.1%)	10 (6.6%)	
	Incidental finding	9 (8.7%)	29 (14.8%)	20 (13.2%)	
Hydronephrosis		60 (57.7%)	116 (59.2%)	79 (52.0%)	0.387
Creatinine (mg/dL)		1.42 ± 1.44	1.46 ± 1.54	1.32 ± 1.20	0.641
Operation time (min)		359.1 ± 96.2	351.7 ± 114.5	339.6 ± 84.6	0.292
Estimated blood loss (cc)		498.9 ± 527.2	272.9 ± 369.9	161.9 ± 226.1	<0.001
**Laterality**					**0.839**
	Right	44 (42.3%)	87 (44.4%)	70 (46.1%)	
	Left	60 (57.7%)	109 (55.6%)	82 (53.9%)	
**Tumor site**					**0.520**
	Renal pelvis	64 (61.5%)	110 (56.1%)	82 (53.9%)	
	Ureter	25 (24.1%)	64 (32.7%)	50 (32.9%)	
	Renal pelvis and ureter	15 (14.4%)	22 (11.2%)	20 (13.2%)	
**Pathology**					**0.852**
	Papillary UC	41 (39.4%)	76 (38.8%)	64 (42.1%)	
	Infiltrating UC	16 (15.4%)	31 (15.8%)	20 (13.2%)	
	Papillary and infiltrating UC	47 (45.2%)	86 (43.9%)	67 (44.0%)	
	CIS only	0	3 (1.5%)	1 (0.7%)	
**Stage**					**0.205**
	Ta + T1	46 (44.2%)	81 (41.3%)	78 (51.3%)	
	T2	10 (9.6%)	19 (9.7%)	20 (13.2%)	
	T3 + T4	48 (46.2%)	93 (47.4%)	53 (34.9%)	
	CIS only	0	3 (1.5%)	1 (0.7%)	
**Grade**					**0.942**
	Low grade	8 (7.7%)	14 (7.1%)	10 (6.6%)	
	High grade	96 (92.3%)	182 (92.9%)	142 (93.4%)	
Tumor size (cm)		3.6 ± 2.2	3.1 ± 1.9	3.2 ± 2.0	0.135
Concomitant CIS		22 (21.2%)	45 (23.0%)	37 (24.3%)	0.837
Multifocality		34 (32.7%)	54 (27.6%)	48 (31.6%)	0.578
Bladder cuff involvement		2 (1.9%)	2 (1.0%)	3 (2.0%)	0.728
Variant histology**		14 (13.5%)	21 (10.7%)	11 (7.2%)	0.256
Lymphovascular invasion		12 (11.5%)	17 (8.7%)	11 (7.2%)	0.489
Positive margin		1 (1.0%)	4 (2.0%)	1 (0.7%)	0.500
Lymph node metastasis		12 (11.5%)	9 (4.6%)	8 (5.3%)	0.051
Systemic adjuvant therapy before IVR		33 (31.7%)	46 (23.5%)	33 (21.7%)	0.162
Intravesical recurrence		24 (23.1%)	69 (35.2%)	41 (27.0%)	0.062

RNU = radical nephroureterectomy; BCE = bladder cuff excision; MIS = minimally invasive surgery; BMI = body mass index; HTN = hypertension; DM = diabetes mellitus; CAD = coronary artery disease; UC = urothelial carcinoma; CIS = carcinoma in situ; IVR = intravesical recurrence

Data are presented as mean ± standard deviation or count (percentage).

* Smoking: ever-smokers

** Variant histology: squamous differentiation, sarcomatoid change, micropapillary pattern, glandular differentiation, lymphoepithelioma-like carcinoma variant, and mixed small cell neuroendocrine carcinoma.

The median duration of follow-up was 29.5 months (interquartile range [IQR]=15.1–52.4 months); it was comparable among the three groups. Of the 452 patients, 134 (29.6%) patients experienced IVR after surgery, and the median time to IVR was 7.6 months (IQR=3.6–13.1 months). A total of 452 patients had IVR-free survival rates of 68.6% 2 years postoperatively and 64.3% 5 years postoperatively.

**Figure 1:**
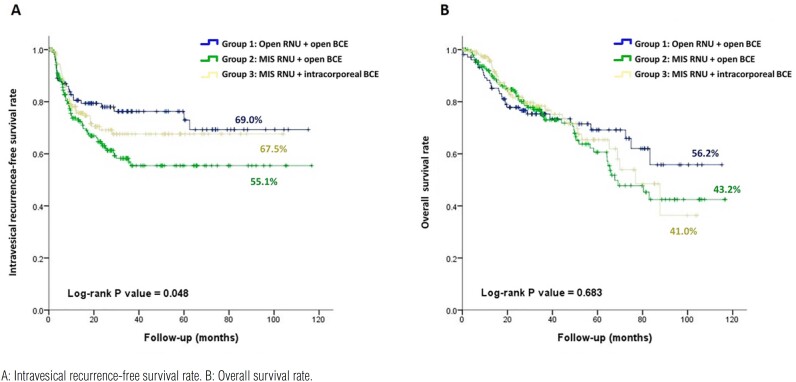
Survival rates in patients with upper tract urothelial carcinoma who received open RNU with open BCE, MIS RNU with open BCE, or MIS RNU with intracorporeal BCE.

Group 2 had a significantly lower IVR-free survival rate than the other groups (group 1 vs. group 2 vs. group 3: 69.0% vs. 55.1% vs. 67.5%; log-rank P=0.048) (Figure-1A). The overall survival rate was not significantly different among the three groups (group 1 vs. group 2 vs. group 3: 56.2% vs. 43.2% vs. 41.0%; log-rank P=0.683) (Figure-1B). No patient in group 2 (0%) underwent robotic procedures, while 123 (80.9%) patients in group 3 underwent robotic procedures. Thus, we performed subgroup analysis by MIS procedures (i.e., laparoscopic vs. robotic approach) only for group 3. There was no significant difference in the IVR-free survival rate (laparoscopic approach vs. robotic approach: 59.6% vs. 69.9%; log-rank P=0.156) and in the overall survival rate (laparoscopic approach vs. robotic approach: 45.2% vs. 43.6%; log-rank P=0.732). Univariate Cox regression analysis revealed that BMI, surgical procedure, tumor site, stage, multifocality, concomitant CIS, variant histology, tumor size, and systemic adjuvant therapy before IVR were significantly associated with IVR (Table-2). In the multivariate analysis, higher BMI (HR=1.778, 95% CI=1.125-2.810, P=0.014), the surgical procedure of MIS RNU with open BCE (HR=1.949, 95% CI=1.082-3.511, P=0.026), and tumors located in the renal pelvis and ureter (HR=2.896, 95% CI=1.332-6.295, P=0.007) were associated with a higher risk of IVR. Systemic adjuvant therapy before IVR was associated with a lower risk of IVR after surgery (HR=0.410, 95% CI=0.206–0.816, P=0.011) (Table-3).

**Table 2:** Univariate Cox regression analysis of the influencing factors of intravesical recurrence after surgery in patients with localized upper tract urothelial carcinoma.

Variable		Hazard ratio	95% CI	P-value
Sex (male vs. female)		1.310	0.874-1.963	0.191
Age (year) (≥ 70 vs. <70)		1.057	0.700-1.596	0.793
BMI (kg/m^2^) (≥ 24 vs. <24)		1.528	1.012-2.308	0.044
Smoking*		1.613	0.976-2666	0.062
**Symptoms**				
	Hematuria	1.0 (Ref)		
	Other symptoms	0.343	0.117-1.010	0.052
	Incidental finding	0.683	0.360-1.296	0.243
Voiding urine cytology (positive vs. others)		1.052	0.645-1.717	0.838
Flushing urine cytology (positive vs. others)		0.963	0.561-1.653	0.890
Preoperative hydronephrosis		0.857	0.571-1.286	0.455
Preoperative Creatinine		1.031	0.898-1.184	0.664
**Surgical procedure**				
	Group 1: Open RNU + open BCE	1.0 (Ref)		
	Group 2: MIS RNU + open BCE	1.811	1.053-3.115	0.032
	Group 3: MIS RNU + intracorporeal BCE	1.231	0.689-2.199	0.482
Side (left vs. right)		0.941	0.627-1.412	0.770
**Tumor site**				
	Renal pelvis	1.0 (Ref)		
	Ureter	1.449	0.917-2.290	0.112
	Renal pelvis and ureter	3.021	1.670-5.465	<0.001
Operation time (min) (≥360 vs. <360)		0.986	0.655-1.484	0.946
Estimated blood loss (mL) (≥300 vs. <300)		0.834	0.540-1.287	0.411
**Pathology**				
	Papillary UC	1.0 (Ref)		
	Infiltrating UC	0.653	0.348-1.225	0.184
	Papillary and infiltrating UC	0.692	0.446-1.074	0.100
	CIS only	1.919	0.264-13.955	0.519
**Stage**				
	Ta + T1 + CIS	1.0 (Ref)		
	T2	0.942	0.486-1.827	0.861
	T3 + T4	0.621	0.402-0.961	0.032
Grade (high vs. low)		1.548	0.653-3.671	0.321
Bladder cuff involvement		3.231	0.713-14.637	0.128
Multifocality		2.462	1.607-3.773	<0.001
Concomitant CIS		2.757	1.747-4.350	<0.001
Variant histology**		0.326	0.135-0.788	0.013
Tumor size (cm) (≥ 2.5 vs. <2.5)		0.652	0.414-0.945	0.026
Lymphovascular invasion		0.667	0.308-1.441	0.303
Lymph node metastasis		0.601	0.239-1.512	0.280
Positive margin		0.471	0.054-4.067	0.493
Systemic adjuvant therapy before IVR		0.431	0.253-0.735	0.002

CI = confidence interval; BMI = body mass index; RNU = radical nephroureterectomy; BCE = bladder cuff excision; MIS = minimally invasive surgery; UC = urothelial carcinoma; CIS = carcinoma in situ; IVR = intravesical recurrence;

* Smoking: ever-smokers;

** Variant histology: squamous differentiation, sarcomatoid change, micropapillary pattern, glandular differentiation, lymphoepithelioma-like carcinoma variant, and mixed small cell neuroendocrine carcinoma.

**Table 3:** Multivariate Cox regression analysis of the independent influencing factors of intravesical recurrence after surgery in patients with localized upper tract urothelial carcinoma.

Variable		Hazard ratio	95% CI	P-value
BMI (kg/m^2^) (≥ 24 vs. <24)		1.778	1.125-2.810	0.014
Smoking*		1.508	0.870-2.616	0.144
**Surgical procedure**				
	Group 1: Open RNU + open BCE	1.0 (Ref)		
	Group 2: MIS RNU + open BCE	1.949	1.082-3.511	0.026
	Group 3: MIS RNU + intracorporeal BCE	1.108	0.591-2.076	0.750
**Tumor site**				
	Renal pelvis	1.0 (Ref)		
	Ureter	1.127	0.681-1.864	0.643
	Renal pelvis and ureter	2.896	1.332-6.295	0.007
**Stage**				
	Ta + T1 + CIS	1.0 (Ref)		
	T2	0.868	0.418-1.803	0.704
	T3 + T4	0.904	0.505-1.618	0.734
Multifocality		1.496	0.714-3.134	0.286
Concomitant CIS		1.674	0.801-3.499	0.171
Variant histology**		0.430	0.167-1.109	0.081
Tumor size (cm) (≥ 2.5 vs. <2.5)		0.715	0.446-1.145	0.163
Systemic adjuvant therapy before IVR		0.410	0.206-0.816	0.011

CI = confidence interval; BMI = body mass index; RNU = radical nephroureterectomy; BCE = bladder cuff excision; MIS = minimally invasive surgery; CIS = carcinoma in situ; IVR = intravesical recurrence

* Smoking: ever-smokers

** Variant histology: squamous differentiation, sarcomatoid change, micropapillary pattern, glandular differentiation, lymphoepithelioma-like carcinoma variant, and mixed small cell neuroendocrine carcinoma.

## DISCUSSION

This study revealed that a combination of MIS RNU and open BCE is associated with a significantly lower IVR-free survival than open RNU with open BCE and MIS RNU with intracorporeal BCE. Notably, the multivariate analysis also revealed that group 2 patients had a significantly higher risk of IVR than groups 1 and 3 patients, while groups 1 and 3 patients had comparable risks of IVR. To the best of our knowledge, this is the first large-scale study (>450 UTUC patients) to compare the oncological outcomes of different combinations of RNU and BCE surgical procedures and investigate the impact of these combinations on IVR.

A completely minimally invasive procedure for UTUC is technically challenging because of the spatial limitations of intracorporeal BCE in the deep pelvic cavity. Various BCE procedures have been proposed and combined with laparoscopic or robotic RNU (2, 17, 18). However, only a few studies have compared the oncological outcomes of these combinations and conducted further multivariate analyses to clarify the therapeutic effects of these procedures. In a study by Zhang et al., 45 patients with UTUC who underwent total laparoscopic RNU with BCE in a single surgical position were compared with 44 patients who underwent retroperitoneal laparoscopic RNU with open BCE. The study reported comparable tumor recurrence rates between the two patient groups, including intravesical, extravesical, renal-pelvic, and ureteral tumor recurrence (19). Miyake et al. also demonstrated complete retroperitoneal laparoscopic RNU with transvesical BCE in four patients with UTUC. They compared the postoperative outcomes with those of retroperitoneal laparoscopic RNU with open BCE performed in four other patients and observed similar postoperative pain between the two groups; however, no oncological outcomes were reported (20). Ye et al. reported that robot-assisted RNU with robotic extravesical BCE (n=29) and laparoscopic RNU with BCE (n=131) provided comparable 5-year IVR-free survival and distant metastasis-free survival and lower but non-significant 5-year retroperitoneal recurrence-free survival and cancer-speciﬁc survival. However, the laparoscopic RNU with BCE group included patients who underwent laparoscopic BCE (n = 66) and open BCE (n = 65). The oncological outcomes of these different combinations could not be further verified (21). Similarly, in a study by Hemal et al., the retroperitoneal laparoscopic RNU group comprised a mix of patients who underwent laparoscopic BCE and open BCE. Although this study had a control group of open RNU with BCE, there was no comparison between different combinations (22).

Contrary to previous reports, our study presents a large, homogenous, and evenly distributed group of patients with UTUC treated with three different surgical combinations of RNU and BCE. Our experiences highlight an important but neglected factor that may influence IVR after RNU with BCE for UTUC. Physicians should take this factor into consideration in surgical planning for UTUC, along with other surgery-specific factors regarding IVR.

This study has some limitations. First, this was a retrospective and nonrandomized study. Therefore, potential selection and reporting biases cannot be avoided. Second, all patients were treated at a single medical center, which limits the external validity of this study. Third, postoperative follow-up protocols for each surgical procedure may vary. The surgeons’ experiences were also not considered. Fourth, none of the included patients received immediate postoperative intravesical chemotherapy instillation. Postoperative intravesical chemotherapy has been reported to be associated with lower bladder recurrence but has not been routinely incorporated into the clinical care of patients with UTUC (23, 24). However, although we did not routinely perform postoperative intravesical therapy, our practice is homogenous which would omit this potentially confounding factor. Finally, we routinely performed ureteroscopic biopsy for the definitive diagnosis of UTUC prior to RNU BCE. Some studies reported that ureteroscopy with or without biopsy may increase the risk of IVR (25, 26). However, the 29.6% IVR rate in our series is not higher than other series that did not routinely perform ureteroscopy for diagnosis (1, 8–12). Meanwhile, ureteroscopic biopsy is our routine practice; thus, no biases were introduced.

## CONCLUSIONS

For patients with UTUC, the IVR-free survival rate is significantly lower in those undergoing MIS RNU with open BCE. MIS RNU with open BCE is significantly and independently associated with a higher risk of IVR than open RNU with open BCE or MIS RNU with intracorporeal BCE. Further studies, including multi-institutional collaboration, are required to validate the results and develop standardized preoperative surgical planning and postoperative follow-up protocols.

## ABBREVIATIONS

UTUC: upper tract urothelial carcinoma
RNU: radical nephroureterectomy
BCE: bladder cuff excision
MIS: minimally invasive surgery
BMI: body mass index
DM: diabetes mellitus
HTN: hypertension
CAD: coronary artery disease
UC: urothelial carcinoma
CIS: carcinoma in situ
IVR: intravesical recurrence
IQR: interquartile range
CI: confidence interval

## References

[1] Roupret M, Babjuk M, Burger M, Capoun O, Cohen D, Compérat EM (2021). European Association of Urology Guidelines on upper urinary tract urothelial carcinoma: 2020 update. Eur Urol.

[2] Macejko AM, Pazona JF, Loeb S, Kimm S, Nadler RB (2008). Management of distal ureter in laparoscopic nephroureterectomy-a comprehensive review of techniques. Urology.

[3] Li WM, Shen JT, Li CC, Ke HL, Wei YC, Wu WJ (2010). Oncologic outcomes following three different approaches to the distal ureter and bladder cuff in nephroureterectomy for primary upper urinary tract urothelial carcinoma. Eur Urol.

[4] Xylinas E, Rink M, Cha EK, Clozel T, Lee RK, Fajkovic H (2014). Impact of distal ureter management on oncologic outcomes following radical nephroureterectomy for upper tract urothelial carcinoma. Eur Urol.

[5] Hora M, Eret V, Urge T, Klečka J, Trávníček I, Hes O (2012). Complete laparoscopic nephroureterectomy with intravesical lockable clip. Cent European J Urol.

[6] Hattori R, Yoshino Y, Komatsu T, Matsukawa Y, Ono Y, Gotoh M (2009). Pure laparoscopic complete excision of distal ureter with a bladder cuff for upper tract urothelial carcinoma. World J Urol.

[7] Sparwasser P, Epple S, Thomas A, Dotzauer R, Boehm K, Brandt MP (2022). First completely robot-assisted retroperitoneal nephroureterectomy with bladder cuff: a step-by-step technique. World J Urol.

[8] Fang D, Li X-S, Xiong G-Y, Yao L, Yao L, He ZS (2013). Prophylactic intravesical chemotherapy to prevent bladder tumors after nephroureterectomy for primary upper urinary tract urothelial carcinomas: a systematic review and meta-analysis. Urol Int.

[9] Zigeuner RE, Hutterer G, Chromecki T, Rehak P, Langner C (2006). Bladder tumour development after urothelial carcinoma of the upper urinary tract is related to primary tumour location. BJU Int.

[10] Novara G, De Marco V, Dalpiaz O, Gottardo F, Bouygues V, Galfano A (2008). Independent predictors of metachronous bladder transitional cell carcinoma (TCC) after nephroureterectomy for TCC of the upper urinary tract. BJU Int.

[11] Xylinas E, Rink M, Margulis V, Karakiewicz P, Novara G, Shariat SF (2012). Multifocal carcinoma In situ of the upper tract is associated with high risk of bladder cancer recurrence. Eur Urol.

[12] Korkes F, Spiess PE, Garcia-Perdomo HA, Necchi A (2022). Challenging dilemmas of low grade, non-invasive bladder cancer: a narrative review. Int Braz J Urol.

[13] Kang CH, Yu TJ, Hsieh HH, Yang JW, Shu K, Huang CC (2003). The development of bladder tumors and contralateral upper urinary tract tumors after primary transitional cell carcinoma of the upper urinary tract. Cancer.

[14] Seisen T, Granger B, Colin P, Léon P, Utard G, Renard-Penna R (2015). A systematic review and meta-analysis of clinicopathologic factors linked to intravesical recurrence after radical nephroureterectomy to treat upper tract urothelial carcinoma. Eur Urol.

[15] Edge S, Byrd DR, Compton CC (2010). AJCC Cancer Staging Handbook.

[16] World Health Organization classification of tumours (2004). Pathology & genetics of tumours of the urinary system and male genital organs.

[17] Cho HJ, Kim SJ, Yoon BI, Yoon BI, Lee JY, Kim SW (2010). A novel bulldog clamp technique for management of a distal ureter and bladder cuff during laparoscopic nephroureterectomy. J Endourol.

[18] Shoma AM (2009). Purse-string technique for laparoscopic excision of a bladder mucosal cuff in patients with transitional cell carcinoma of the upper urinary tract: initial report with intermediate follow-up. BJU Int.

[19] Zhang X, Wang K, Ma J, Zhang Q, Liu C, Cui Y (2019). Total laparoscopic nephroureterectomy for upper urinary tract urothelial carcinoma under a single surgical position. World J Surg Oncol.

[20] Miyake M, Nishimura N, Aoki K, Ohmori C, Shimizu T, Owari T (2020). Initial experience of complete laparoscopic radical nephroureterectomy combined with transvesical laparoscopic excision of distal ureter in patients with upper urinary tract cancer. World J Surg Oncol.

[21] Ye H, Feng X, Wang Y, Zhang C, Zhang W, Guo F (2020). Single-docking robotic-assisted nephroureterectomy and extravesical bladder cuff excision without intraoperative repositioning: The technique and oncological outcomes. Asian J Surg.

[22] Hemal AK, Kumar A, Gupta NP, Seth A (2008). Retroperitoneal nephroureterectomy with excision of cuff of the bladder for upper urinary tract transitional cell carcinoma: comparison of laparoscopic and open surgery with long-term follow-up. World J Urol.

[23] Fan B, Teng Q, Sun M, Wang Y, Wang Y, Lin Z (2022). Assessment of therapeutic benefit and option strategy on intravesical instillation for preventing bladder cancer recurrence after radical nephroureterectomy in patients with upper urinary tract urothelial carcinoma. J Oncol.

[24] Del Giudice F, van Uem S, Li S, Vilson FL, Sciarra A, Salciccia S (2022). Contemporary trends of systemic neoadjuvant and adjuvant intravesical chemotherapy in patients with upper tract urothelial carcinomas undergoing minimally invasive or open radical nephroureterectomy: analysis of US claims on perioperative outcomes and health care costs. Clin Genitourin Cancer.

[25] Sharma V, Miest TS, Juvet TS, Toussi A, Packiam V, Chamie K The impact of upper tract urothelial carcinoma diagnostic modality on intravesical recurrence after radical nephroureterectomy: a single institution series and updated meta-analysis. J Urol.

[26] Nowak Ł, Krajewski W, Chorbinska J, Kiełb P, Sut M, Moschini M (2021). The impact of diagnostic ureteroscopy prior to radical nephroureterectomy on oncological outcomes in patients with upper tract urothelial carcinoma: a comprehensive systematic review and meta-analysis. J Clin Med.

